# Non-Obstructive Accessory Mitral Valve Tissue in an Asymptomatic Adult: A Case Report and Review of Literature

**DOI:** 10.7759/cureus.10340

**Published:** 2020-09-09

**Authors:** Rupinder Buttar, Bipul Baibhav

**Affiliations:** 1 Cardiology, Rochester Regional Health, Rochester, USA; 2 Cardiology, Sands-Constellation Heart Institute, Rochester Regional Health, Rochester, USA

**Keywords:** accessory mitral valve tissue, lvoto, transthoracic echocardiogram, left ventricular outflow tract obstruction

## Abstract

Accessory mitral valve tissue (AMVT) is a rare congenital cardiac anomaly that is often an asymptomatic incidental finding. However, it has also been reported to be an important cause of left ventricular outflow tract obstruction (LVOTO) in subset of patients. When symptomatic, patients can often present with symptoms, including dyspnea, chest pain and palpitations/arrhythmias. Surgical resection is indicated in symptomatic cases with significant LVOTO. We here report a 50-year-old male who presented with chest pain and was incidentally found to have AMVT on an echocardiogram. No evidence of LVOTO was seen at rest, Valsalva, or stress. We also provide a review of literature in regards to most relevant clinical implication of AMVT.

## Introduction

Accessory mitral valve tissue (AMVT) is a rare congenital anomaly with a reported incidence of 1:26,000, mostly diagnosed in the first decade of life [1]. It may also be associated with various other congenital intracardiac and vascular malformations, such as atrial and ventricular septal defects. When detected in adulthood, it is often an incidental finding on an echocardiogram [2]. However, it can also be an important cause of left ventricular outflow tract obstruction (LVOTO). If symptomatic, treatment of choice is usually surgical resection [3]. We here present a case of an asymptomatic patient with incidental finding of AMVT on echocardiogram. 

## Case presentation

A 50-year-old male with past medical history significant for hypertension presented to the emergency room with chief complaint of chest pain. He described the chest pain as sharp, non-radiating, midsternal with no alleviating or exacerbating factors. He denied any exertional chest pain, dyspnea, palpitations or syncope. His home medication included hydrochlorothiazide 25 mg daily. The patient admitted to non-compliance to his home medication. Family history was unremarkable for any cardiac disease or sudden cardiac death. He denied history of smoking, alcohol use or drug use.

Physical examination was significant for elevated blood pressure of 169/88 mm Hg. Physical examination was normal with no murmurs or gallops. Electrocardiogram (EKG) showed normal sinus rhythm with non-specific T-wave inversions in lead V4-V6 and lead III and no evidence of left ventricular hypertrophy or pathological Q waves. Laboratory testing revealed normal blood count, renal function tests and troponin levels. 

The patient had a treadmill exercise stress echocardiogram for further cardiac evaluation. Resting cardiac echocardiogram showed normal cardiac chamber size, wall thickness and systolic function. The ejection fraction was noted to be 65%, and there were no regional wall motion abnormalities. Accessory anterior mitral leaflet tissue was noted, which prolapsed into the left ventricular outflow tract (LVOT) during systole (Video 1).

**Video 1:** Accessory anterior mitral leaflet tissue noted, which prolapses into the left ventricular outflow tract (LVOT) during systole

There was no evidence of dynamic LVOT at rest, Valsalva or stress (Video 2).

**Video 2:** No evidence of dynamic left ventricular outflow tract (LVOT) seen

No associated mitral regurgitation was seen. Stress echocardiogram did not show any evidence of inducible wall motion abnormalities to suggest ischemia, and there were no EKG changes of ischemia. As AMVT did not cause any symptoms of LVOTO, a decision was made to follow the patient without any intervention.

## Discussion

First reported in 1842, AMVT is a rare congenital cardiac anomaly. Although the exact origin is unknown, it is considered to arise as a result of incomplete separation of mitral valve from the endocardial cushions or as a remnant of endocardial cushions that did not fuse with the mitral valve leaflet [4,5]. It can be associated with various other congenital abnormalities, including ventricular septal defect, atrial septal defect, subaortic stenosis, transposition of great arteries, coronary artery anomalies and coarctation of aorta.

Based on the mobility, it has been classified into two main types by Prifiti et al: fixed and mobile. This is further subdivided based on insertion, relationship to the mitral valve and development of chordae. The most common presentation is type IIB leaflet form, located on ventricular side of the anterior mitral valve leaflet [4,6]. 

Although AMVT may remain asymptomatic in patients, it may lead to LVOTO in some patients. As suggested by Manganaro et al. based on previous literature, the symptoms usually present when the LVOT gradient is ≥50 mm Hg [4]. Interestingly, one-third of the patients with LVOTO present within the first decade of life. The most common symptoms are usually dyspnea followed by chest pain and palpitations/arrhythmias. Possible association between AMVT and transient ischemic attack (TIA) has also been reported in few case reports. As AMVT may also be associated with other congenital heart abnormalities, the symptoms and presentations can vary.

Echocardiography is considered the “gold standard” modality for the evaluation of AMVT. A transthoracic echocardiogram (TTE) can not only help diagnose this condition but can also detect associated congenital heart defects. Transesophageal echocardiogram (TEE) can be considered for further characterization of morphology, shape and attachments of AMVT. Other modalities such as cardiac magnetic resonance can also be used. 

Beta-blocker therapy can be tried in patients with AMVT who have significant degree of LVOTO at rest or with provocation [5]. Primary cardiac intervention for symptomatic patients with AMVT and LVOTO is surgical resection. This may be indicated in symptomatic patients with significant degree of LVOTO. It can also be performed as part of other cardiac surgery, including those undergoing repairs of congenital cardiac abnormalities, commonly seen with AMVT. Surgical technique involves excision of the AMVT along with the division of the accessory chordae. It is a relatively safe procedure with postop mortality of 8.9% largely driven by incomplete removal of AMVT and associated congenital cardiac defects [4,6]. 

Data regarding follow-up surveillance in asymptomatic patients are still lacking with most case series reporting an annual follow-up.

## Conclusions

AMVT is a rare congenital cardiac anomaly that is usually an asymptomatic incidental finding; however, it may present with symptoms of LVOTO. When symptomatic, it is usually managed by surgical resection. Limited data are available regarding surveillance and anticoagulation in asymptomatic patients. 

## References

[1] Okafor J, Kanaganayagam GS, Patel K (2020). A rare finding of giant accessory mitral valve tissue: a case report. Eur Heart J Case Rep.

[2] Yuan SM, Shinfeld A, Mishaly D, Haizler R, Ghosh P, Raanani E (2008). Accessory mitral valve tissue: a case report and an updated review of literature. J Card Surg.

[3] Panduranga P, Eapen T, Al-Maskari S, Al-Farqani A (2011). Accessory mitral valve tissue causing severe left ventricular outflow tract obstruction in a post-Senning patient with transposition of the great arteries. Heart Int.

[4] Manganaro R, Zito C, Khandheria BK (2014). Accessory mitral valve tissue: an updated review of the literature. Eur Heart J Cardiovasc Imaging.

[5] Al-Atta A, Khan H, Sosin M (2019). Accessory mitral valve tissue causing features of left ventricular outflow tract obstruction: a case report and updated literature review. J Ayub Med Coll Abbottabad.

[6] Prifti E, Bonacchi M, Bartolozzi F, Frati G, Leacche M, Vanini V (2003). Postoperative outcome in patients with accessory mitral valve tissue. Med Sci Monit.

